# The survival analysis of rifampicin/multidrug-resistant tuberculosis patients based on the levels of inflammatory biomarkers: a retrospective cohort study

**DOI:** 10.3389/fcimb.2023.1118424

**Published:** 2023-05-01

**Authors:** Qi Yu, Hong Luo, Shengling Hu, Dan Sun, Qi Nie, Jisong Yan

**Affiliations:** ^1^ Department of Infectious Diseases, Wuhan Jinyintan Hospital, Tongji Medical College of Huazhong University of Science and Technology, Wuhan, Hubei, China; ^2^ Department of Respiratory and Critical Care Medicine, Wuhan Jinyintan Hospital, Tongji Medical College of Huazhong University of Science and Technology, Wuhan, Hubei, China; ^3^ Department of Interventional Therapy, Wuhan Pulmonary Hospital, Wuhan, Hubei, China; ^4^ Department of MDR/RR-TB, Wuhan Jinyintan Hospital, Tongji Medical College of Huazhong University of Science and Technology, Wuhan, Hubei, China

**Keywords:** rifampicin/multidrug-resistant tuberculosis, prognosis, inflammatory markers, clincial features, inflammation

## Abstract

**Purpose:**

The development of tuberculosis and inflammatory status are closely related. The aim of this study was to investigate the prognostic value of inflammatory biomarkers in patients with rifampicin/multidrug-resistant tuberculosis (RR/MDR-TB).

**Patients and methods:**

This study recruited 504 patients with RR/MDR-TB from Wuhan Jinyintan Hospital. A total of 348 RR/MDR patients from January 2017 to December 2019 were defined as training set, the rest of patients as validation set. The patients were divided into three-risk degrees according to the levels of inflammatory biomarkers (median, 85th percentile). Kaplan-Meier curve and log-rank test were used to assess survival differences among the groups. Cox proportion risk regression was used to identify risk factors for RR/MDR-TB mortality.

**Results:**

In training set, cox proportion risk regression analysis showed that high age (≥60 years) [OR (95%CI):1.053(1.03188-1.077)], smoking [OR (95%CI):2.206(1.191-4.085)], and bronchiectasia [OR (95%CI):2.867(1.548-5.311)] were prognostic factors for RR/MDR-TB patients. In addition, lower survival rates were observed in high CAR group [OR (95%CI):1.464(1.275-1.681)], high CPR group[OR (95%CI):1.268(1.101-1.459)], high CLR group[OR (95%CI):1.004(1.002-1.005)], high NLR group[OR (95%CI):1.103(1.069-1.139)], high PLR group[OR (95%CI):1.003(1.002-1.004)], and high MLR group[OR (95%CI):3.471(2.188-5.508)].Furthermore, AUCs of age, smoking, bronchiectasia, CAR, CPR, CLR, NLR, PLR, and MLR for predicting mortality in RR/MDR-TB patients were 0.697(95%CI:0.618-0.775), 0.603(95%CI:0.512-0.695), 0.629(95%CI:0.538-0.721), 0.748(95%CI:0.675-0.821, P<0.05), 0.754(95%CI:0.683-0.824, P<0.05), 0.759(95%CI:0.689-0.828, P<0.05), 0.789(95%CI:0.731-0.846, P<0.05), 0.740(95%CI:0.669-0.812, P<0.05), and 0.752(95%CI:0.685-0.819, P<0.05), respectively. Importantly, the AUC of predicting mortality of combination of six inflammatory biomarkers [0.823 (95%CI:0.769-0.876)] is higher than any single inflammatory biomarkers. Additionally, the similar results are also obtained in the validation set.

**Conclusion:**

Inflammatory biomarkers could predict the survival status of RR/MDR-TB patients. Therefore, more attention should be paid to the level of inflammatory biomarkers in clinical practice.

## 1 Introduction

Tuberculosis (TB) is a global health concern with high morbidity and mortality. Compared to drug-sensitive TB (DS-TB), RR/MDR-TB typically has longer treatment duration, poorer treatment outcomes, and a higher risk of mortality (Chung-Delgado et al., 2015). According to the World Health Organization (WHO) Global Tuberculosis Report 2021, mortality among RR/MDR-TB patients had increased worldwide with the COVID-19 pandemic (World Health, 2021).

Currently, an increasing number of studies are focused on finding valid markers to predict mortality and exploring risk factors for death in TB patients to improve treatment and reduce mortality, especially in RR/MDR-TB patients. Obviously, Scholars had made some progress in it. It was reported that extrapulmonary TB, age, sex, weight, and comorbidities such as diabetes mellitus (DM) and HIV strongly influence the prognosis of TB (Balaky et al., 2019; Limenih and Workie, 2019; Xie et al., 2020; Machmud et al., 2021). For instance, patients with higher age or extrapulmonary TB tended to have higher mortality (Balaky et al., 2019), and significantly lower survival rates were observed in MDR-TB patients with HIV infection (Machmud et al., 2021). However, previous studies have largely concentrated on clinical features, and few on inflammatory biomarkers. In fact, the inflammatory state was closely related to the development and progression of TB. The release of inflammatory factors along with the formation of an inflammatory microenvironment provided favorable conditions for tissue destruction, bacterial transmission (Amaral et al., 2021), and development and progression of TB (Krug et al., 2021). Fausto Ciccacci’s colleagues and himself reported that high-sensitivity C-reactive protein could predict early mortality in HIV-positive TB patients (Ciccacci et al., 2021).

Although the clinical characteristics associated with mortality in TB patients have been extensively studied, their actual prognostic value was unsatisfactory due to low predictive accuracy, which may limit their clinical application. Therefore, the discovery of novel reliable prognostic biomarkers for patients with RR/MDR-TB is essential for individualized treatment and improved survival. Inexpensive and readily available inflammatory biomarkers, such as C-reactive protein to albumin ratio (CAR), C-reactive protein to prealbumin ratio (CPR), C-reactive protein to lymphocyte ratio (CLR), neutrophil to lymphocyte ratio (NLR), platelet to lymphocyte ratio (PLR) and monocyte to lymphocyte ratio (MLR), have been widely described in various diseases to predict prognosis, especially in malignant tumors. These inflammatory markers may be superior to clinical features for mortality prediction in RR/MDR-TB patients since they exhibit better prognostic value in malignant tumors than clinical features such as TNM classification (Miura et al., 2021).

This study attempted to investigate the effect of inflammatory biomarkers on the prognosis of patients with RR/MDR-TB during anti-tuberculosis treatment (ATT).

## 2 Materials and methods

### 2.1 Participants

We retrospectively collected 504 patients diagnosed with RR/MDR-TB from January 2015 to December 2019 at the Wuhan Jinyintan Hospital, Tongji Medical College, Huazhong University of Science and Technology (Infectious Disease Hospital). Smear microscopy, mycobacterium tuberculosis culture, or GeneXpert MTB/RIF were used for TB diagnosis. RR/MDR-TB was confirmed by drug susceptibility testing (DST). Inclusion criteria were as follows: (1) positive Smear microscopy, mycobacterium tuberculosis culture, or GeneXpert MTB/RIF results; (2) At least rifampin resistance. (3) aged 18 years or older. Exclusion criteria were as follows: (1) dead before treatment; (2) infection of non-tuberculous; (3) refusal of ATT.

### 2.2 Study method and design

1. Division of data set: to ensure the reproducibility of the results, the data of RR/MDR patients from January 2017 to December 2019 in Wuhan Jinyintan Hospital were defined as the training set. And the data from January 2015 to December 2016 (validation set) were applied to validate the results of the training set.
2. Follow-up: The 24-month follow-up was performed for all RR/MDR-TB patients, and the primary outcome of interest was mortality. Censoring was defined as stopping treatment (including cured, completed treatment, treatment failure) or loss to follow-up. The survival time of RR/MDR-TB patients was defined as the date of diagnosis to the date of death, stopping treatment, or loss to follow-up. Definitions of death, cure, completion of treatment, treatment failure, and loss to follow-up are provided in **Supplementary Materials**.
3. Evaluation and validation of prognostic value: based on the 24-month follow-up results, the RR/MDR-TB patients were divided into two groups, namely mortality group and survival group. RR/MDR patients in survival group were defined as stopping treatment, or loss to follow-up.

In the training set, student’s t-test was used to analyze the difference of the inflammatory biomarkers between mortality group and survival group. Cox proportion risk regression was used for investigating risk factors of mortality. Additionally, all patients were risk stratified into three groups according to the levels of inflammatory markers (median and 85th percentile): high-risk group, middle-risk group and low-risk group. Among groups, Kaplan-Meier curve and log-rank test were used for survival analyses. Receiver operating characteristic (ROC) curve was used for assessing the predictive value of the inflammatory biomarkers on prognosis of RR/MDR-TB patients. To further investigate the prognostic value of inflammatory biomarkers, we combined the six inflammatory biomarkers to assess the predictive power of mortality by calculating their AUCs. All analyses were validated in the validation set.

### 2.3 Data processing

The baseline demographics and clinical data (such as radiological findings, laboratory test results, symptoms, treatment history, et al.) were collected through the electronic medical record system (EMRS). The follow-up outcomes were obtained from the national electronic case registry. Inflammatory biomarkers were calculated according to the following formula: CAR: C-reactive protein (mg/L) to albumin (g/L) Ratio; CPR: C-reactive protein (mg/L) to prealbumin (mg/L) ratio; CLR: C-reactive protein (mg/L) to lymphocytes (*10^9/L) ratio; NLR: neutrophils (*10^9/L) to lymphocytes (*10^9/L) ratio; PLR: platelets (*10^9/L) to lymphocytes (*10^9/L) ratio; MLR: monocytes (*10^9/L) to lymphocytes (*10^9/L) ratio.

### 2.4 Statistical analysis

Variables were presented as number with percentage (categorical variables), or mean ± standard deviation (SD)/median (interquartile range, IQR) (continuous variables). Among-groups, differences of continuous variables were compared using student’s t-test or one-way analysis of variance (ANOVA), while categorical variables were assessed by chi-square test or Fisher’s exact test. Cox proportional risk regression was used to investigate risk factors for mortality of patients with RR/MDR-TB. Kaplan-Meier curve and the log-rank test were used for survival analysis. Receiver operating characteristic (ROC) curve was used to assess the predictive value of inflammatory biomarkers on the prognosis of RR/MDR-TB patients.

All statistical analyses were performed by RStudio software (version 4.20). The main R packages included “survival” package, “survminer” package, and “ROCR” package. Two-sided P<0.05 was considered statistically significant.

## 3 Results

### 3.1 Study population

A total of 504 RR/MDR-TB patients diagnosed and treated at the Wuhan Jinyintan Hospital from January 2015 to December 2019 were enrolled in this study. There were 348 patients in the training set and 156 patients in the validation set. As shown in the **Table 1**, treatment outcomes and drug sensitivity have a significant difference among two sets. Mortality rates and XDR-TB percentages are declining over time, meaning that the management and treatment of DS-TB are improving compared to the past. There are no differences between training and validation sets in terms of age, gender, follow-up time, smoking, drinking, pulmonary imaging, underlying condition or illness, and inflammatory biomarkers, suggesting that the data from the training set and validation sets are comparable and can be used for mutual validation. Additionally, **Supplementary Tables 1-6** showed the baseline demographic and clinical characteristics in three risk-degrees of inflammatory biomarkers in training set.

**Table 1:** Demographic, clinical, and laboratory characteristics of the training set and validation set.

Characteristic		Training set				Validation set			
	All (N=348)	Mortality group	Survival group	P value	All (N=156)	Mortality group	Survival group	P value	P value
Age (Mean ± SD)	42.79 ± 15.45	52.76 ± 14.23	41.42 ± 15.12		41.48 ± 15.00	47.5 ± 13.14	39.93 ± 15.10	0.007	0.369
Gender									
Female	84(75.9%)	6	78		48(30.8%)	4	44		
Male	264(24.1%)	36	228	0.112	108(69.2%)	28	80	0.012	0.118
Follow-up time (Mean ± SD)	19.25 ± 7.32	8.92 ± 7.36	20.19 ± 5.21	<0.001	17.88 ± 7.30	7.32 ± 5.08	20.89 ± 5.93	<0.001	0.052
Treatment outcomes									
Cure	122(35.0%)	/	122(35.0%)	/	32(20.5%)	/	32(20.5%)	/	
Treatment completed	119 (34.2%)	/	119 (34.2%)	/	53(34.0%)	/	53(34.0%)	/	
Loss to follow-up	15 (4.3%)	/	15 (4.3%)	/	23(14.7%)	/	23(14.7%)	/	
Failure	50 (14.4%)	/	50 (14.4%)	/	16(10.3%)	/	16(10.3%)	/	
Death	42(12.1%)	42(12.1%)	/	/	32(20.5%)	32(20.5%)	/	/	<0.001
Drug sensitivity									
RR-TB	69(19.8%)	9	60		12(7.7%)	1	11		
MDR-TB	108(31.1%)	13	95		56(35.9%)	5	51		
XDR-TB	171(49.1%)	20	151	0.959	88(56.4%)	26	62	0.006	0.003
Smoking	144(41.4%)	25	119	0.011	59(37.8%)	20	39	0.001	0.451
Drinking	52(14.9%)	12	40	0.008	27(17.3%)	10	17	0.019	0.500
Pulmonary imaging									
Bronchiectasia	127(36.5%)	23	103	0.001	46(29.5%)	18	28	<0.001	0.111
Pulmonary cavity	233(67.0%)	35	198	0.016	96(61.5%)	28	68	0.001	0.238
Pleural effusion	54(15.5%)	11	43	0.042	29(18.6%)	13	16	<0.001	0.390
Underlying condition or illness									
Diabetes mellitus	52(14.9%)	8	44	0.426	25(16.0%)	7	18	0.312	0.755
HIV infection	11(3.2%)	3	8	0.116	2(1%)	1	1	0.299	0.219
Inflammatory biomarkers									
CAR	1.14 ± 1.82	2.25 ± 1.98	0.99 ± 1.27	<0.001	1.42 ± 1.82	2.55 ± 2.63	1.13 ± 1.14	<0.001	0.092
CPR	0.46 ± 0.92	0.92 ± 1.07	0.39 ± 0.88	<0.001	0.51 ± 0.72	1.00 ± 0.95	0.38 ± 0.59	<0.001	0.496
NLR	5.14 ± 5.12	9.33 ± 8.58	4.57 ± 4.15	<0.001	5.84 ± 5.27	10.27 ± 8.16	4.70 ± 3.43	<0.001	0.166
PLR	261.10 ± 178.27	407.80 ± 281.41	240.97 ± 148.81	<0.001	290.98 ± 207.56	401.84 ± 336.26	262.38 ± 147.55	0.010	0.120
MLR	0.50 ± 0.34	0.76 ± 0.48	0.46 ± 0.30	<0.001	0.53 ± 0.34	0.75 ± 0.49	0.48 ± 0.26	<0.001	0.310
CLR	46.29 ± 83.68	96.16 ± 114.92	39.44 ± 76.16	<0.001	55.01 ± 79.09	107.29 ± 113.58	41.52 ± 61.12	<0.001	0.262

### 3.2 Risk assessment of clinical features and inflammatory biomarkers in RR/MDR-TB patients in training set and validation set

In training set, age[OR(95%CI):1.053(1.03188-1.077), P<0.05], smoking [OR(95%CI):2.206(1.191-4.085), P<0.05], drinking [OR(95%CI):2.581(1.321-5.403), P<0.05], bronchiectasia [OR(95%CI):2.867(1.548-5.311), P<0.05], pulmonary cavity [OR(95%CI):2.610(1.159-5.877), P<0.05], and pleural effusion [OR(95%CI):2.028(1.019-4.035)] were risk factors for mortality in RR/MDR-TB patients (**Table 2**). In addition, inflammatory biomarkers also had prognostic value in RR/MDR-TB patients (**Table 2**), such as CAR [OR (95% CI): 1.464 (1.275-1.681), P<0.05], CPR [OR (95% CI): 1.268 (1.101-1.459), P<0.05], CLR [OR (95% CI): 1.004 (1.002-1.005), P<0.05], NLR [OR (95% CI): 1.103 (1.069-1.139), P<0. 05], PLR [OR (95% CI): 1.003 (1.002-1.004), P<0.05], and MLR [OR (95%CI): 3.471 (2.188-5.508), P<0.05]. Importantly, the results of the validation set were consistent with those of the training set (**Supplementary Table 7**).

**Table 2:** Cox proportion risk regression analysis in RR/MDR-TB patients in the training set.

Parameters	P value	OR	95%CI	
			down	Up
Age	<0.001	1.053	1.031	1.077
Sex	0.131	1.947	0.820	4.621
Smoking	0.012	2.206	1.191	4.085
Drinking	0.006	2.581	1.321	5.043
Bronchiectasia	0.001	2.867	1.548	5.311
Pulmonary cavity	0.020	2.610	1.159	5.877
Pleural effusion	0.044	2.028	1.019	4.035
Diabetes mellitus	0.383	1.409	0.652	3.045
HIV infection	0.131	2.470	0.763	7.993
CAR	<0.001	1.464	1.275	1.681
CPR	<0.001	1.268	1.101	1.459
CLR	0.001	1.004	1.002	1.005
NLR	<0.001	1.103	1.069	1.139
PLR	<0.001	1.003	1.002	1.004
MLR	<0.001	3.471	2.188	5.508

CAR, C-reactive protein to Albumin Ratio; CPR, C-reactive protein to Prealbumin Ratio; CLR, C-reactive protein to Lymphocyte Ratio; NLR, Neutrophils to Lymphocyte Ratio; PLR, Platelet to Lymphocyte Ratio; MLR, Monocyte to Lymphocyte Ratio.

### 3.3 Risk stratification in RR/MDR-TB patients in training set

**Figure 1:**
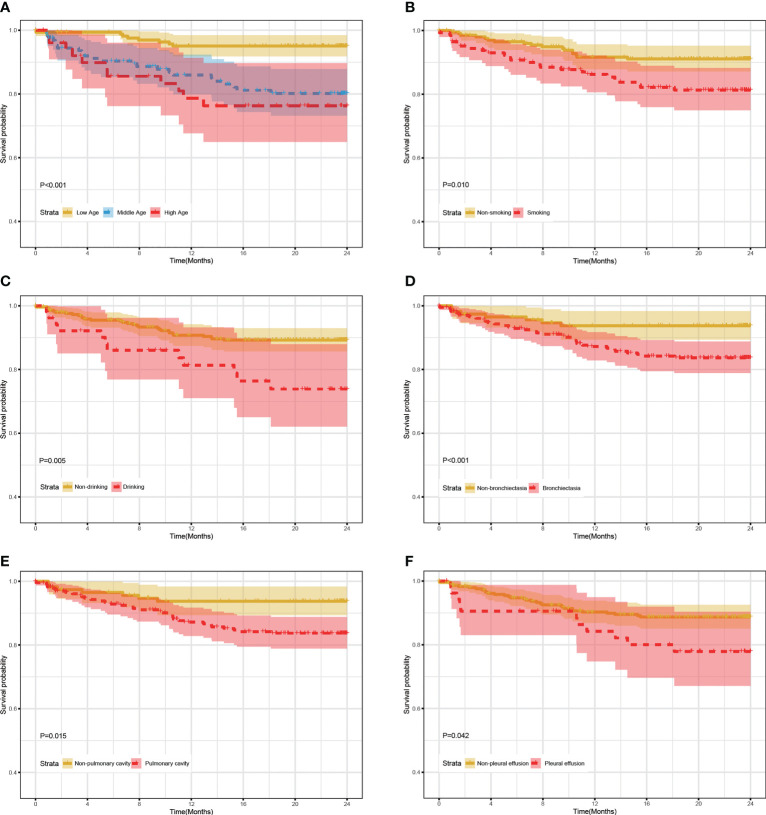
24-month survival rate of RR/MDR-TB patients according to the levels of clinical characteristics in training set. **(A)** The survival analysis according to age. **(B)** The survival analysis according to smoking. **(C)** The survival analysis according to Drinking. **(D)** The survival analysis according to Bronchiectasia. **(E)** The survival analysis according to Pulmonary cavity. **(F)** The survival analysis according to Pleural effusion.

As shown in **Figure 1**, mortality rates were significantly higher in the bronchiectasia, pulmonary cavity, pleural effusion, smoking, and drinking groups than those in the non-exposure groups (log-rank test, all P value < 0.05). In addition, the mortality rate in the middle/low age group was significantly lower than that in the high age group (>60 years old) (log-rank test, all P value < 0.05).

**Figure 2:**
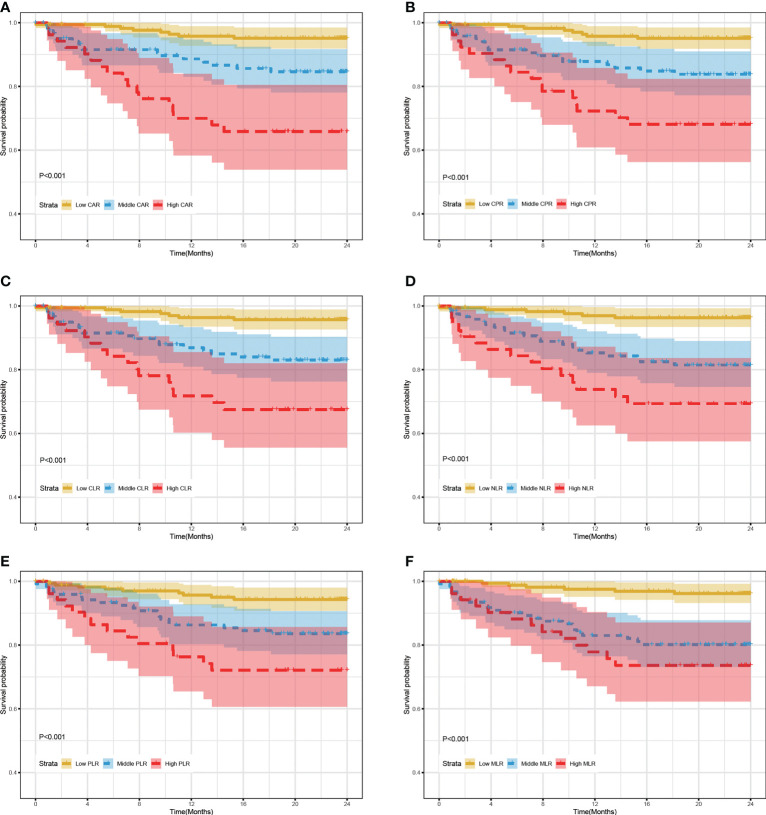
24-month survival rate of RR/MDR-TB patients according to risk stratification of inflammatory biomarkers in training set. **(A)** The survival analysis according to risk stratification of CAR. **(B)** The survival analysis according to risk stratification of CPR. **(C)** The survival analysis according to risk stratification of CLR. **(D)** The survival analysis according to risk stratification of NLR. **(E)** The survival analysis according to risk stratification of PLR. **(F)** The survival analysis according to risk stratification of MLR.

Based on inflammatory biomarkers level (median and 85th percentile), patients with RR/MDR-TB were classified into three risk groups. The cut-off value for each inflammatory biomarker were detailed in the **Supplementary Table 8**. **Figure 2** showed that the mortality rates in the high-risk group, including CAR (A), CPR (B), CLR (C), NLR (D), PLR (E), and MLR (F), were significantly higher than those in the middle/low-risk group (log-rank test, all P value < 0.01). Likewise, the middle-risk group has a higher mortality rate than in the low-risk group (log-rank test, all P value < 0.01).

### 3.4 Predictive value of clinical features and inflammatory biomarkers on prognosis in RR/MDR-TB patients in training set

**Figure 3:**
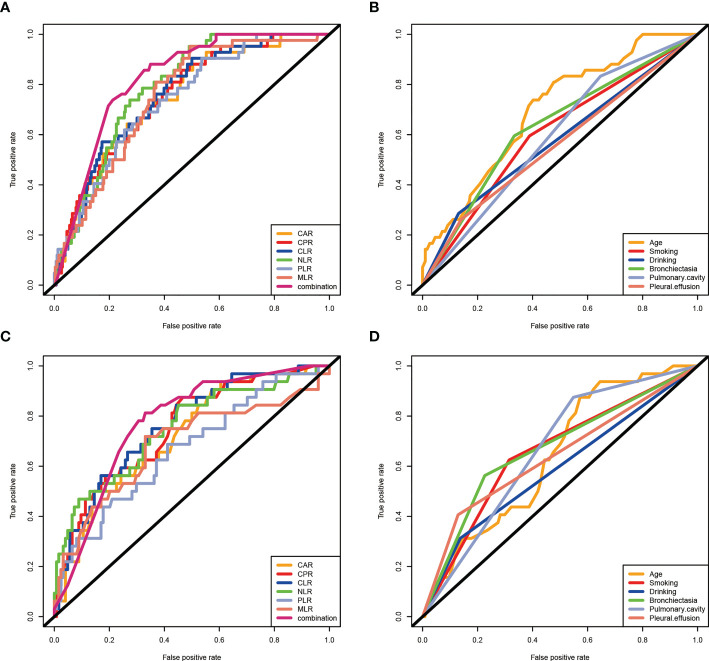
The predictive value of clinical characteristics and inflammatory biomarkers on prognosis in RR/MDR-TB patients. **(A)** Clinical characteristics in training set. The area under the ROC curve (AUC) values in the age, smoking, drinking, bronchiectasia, pulmonary cavity, and pleural effusion were 0.697(95%confidence interval: 0.618-0.775, P<0.05), 0.603(95% confidence interval: 0.512-0.695, P<0.05), 0.577(95%confidence interval: 0.479-0.676, P>0.05), 0.629(95%confidence interval: 0.538-0.721, P<0.05), 0.593(95%confidence interval: 0.508-0.678, P>0.05), and 0.516 (95%confidence interval: 0.463-0.658, P>0.05), respectively. **(B)** Inflammatory markers in training set. The area under the ROC curve (AUC) values in the CAR, CPR, CLR, NLR,PLR, MLR, and combination were 0.748(95%confidence interval: 0.675-0.821, P<0.05), 0.754(95% confidence interval: 0.683-0.824, P<0.05), 0.759(95%confidence interval: 0.689-0.828, P<0.05), 0.789(95%confidence interval: 0.731-0.846, P<0.05), 0.740(95%confidence interval: 0.669-0.812, P<0.05), 0.752(95%confidence interval: 0.685-0.819, P<0.05), and 0.823(95%confidence interval: 0.769-0.876, P<0.05), respectively. **(C)** Clinical features in validation set. The area under the ROC curve (AUC) values in the age, smoking, drinking, bronchiectasia, pulmonary cavity, and pleural effusion were 0.642(95%confidence interval: 0.544-0.739, P<0.05), 0.655(95% confidence interval: 0.547-0.764, P<0.05), 0.588(95%confidence interval: 0.471-0.705, P>0.05), 0.668(95%confidence interval: 0.557-0.779, P<0.05), 0.663(95%confidence interval: 0.567-0.760, P>0.05), and 0.639(95%confidence interval: 0.522-0.755, P>0.05), respectively. **(D)** Inflammatory markers in validaiton set. The area under the ROC curve (AUC) values in the CAR, CPR, CLR, NLR,PLR, MLR, and combination were 0.722(95%confidence interval: 0.627-0.816, P<0.05), 0.750(95% confidence interval: 0.657-0.843, P<0.05), 0.760(95%confidence interval: 0.671-0.850, P<0.05), 0.749(95%confidence interval: 0.649-0.849, P<0.05), 0.667(95%confidence interval: 0.561-0.774, P<0.05), and 0.686(95%confidence interval: 0.571-0.850, P<0.05), 0.771(95%confidence interval:0.674-0.869, P<0.05), respectively.

To further explore the predictive value of clinical features and inflammatory markers in RR/MDR-TB prognosis, ROC curve analysis was performed. **Figure 3A** showed that the AUCs of age, smoking, and bronchiectasia were 0.697(95%CI:0.618-0.775, P<0.05), 0.603(95%CI:0.512-0.695, P<0.05), 0.629(95%CI:0.538-0.721, P<0.05), respectively. Unfortunately, pulmonary cavity, pleural effusion, and drinking had poor predictive value (P>0.05). In addition, the AUCs of CAR, CPR, CLR, NLR, PLR, and MLR were 0.748(95%CI:0.675-0.821, P<0.05), 0.754(95%CI:0.683-0.824, P<0.05), 0.759(95%CI:0.689-0.828, P<0.05), 0.789(95%CI:0.731-0.846, P<0.05), 0.740(95%CI:0.669-0.812, P<0.05), 0.752(95%CI:0.685-0.819, P<0.05), respectively (**Figure 3B**). These results indicated that inflammatory biomarkers had a better predictive value than clinical features, and could be used as prognostic biomarkers of RR/MDR-TB patients. Importantly, the AUC for the combination of the inflammatory biomarkers (AUC: 0.823, 95% CI: 0.769-0.876, P<0.05) was higher than that of any inflammatory biomarkers, which indicated a more accurate prediction (**Figure 3B**).

### 3.5 Predictive value of clinical features and inflammatory biomarkers for mortality in RR/MDR-TB patients in validation set

The results of the prognostic analysis in the validation set were similar to those in the training set. Bronchiectasia, pulmonary cavity, pleural effusion, smoking, and drinking, age, and the different risk-degrees of inflammatory biomarkers also reflected well the prognosis of RR/MDR-TB patients (**Supplementary Figures 1** and **2**). Furthermore, single inflammatory biomarkers and combined inflammatory biomarkers also presented good predictive power in the validation set, as evidenced by AUCs of 0.722(CAR:95%CI:0.627-0.816), 0.750(CPR:95%CI:0.657-0.843), 0.760(CLR:95%CI:0.671-0.850), 0.749(NLR:95%CI:0.649-0.849), 0.667(CAR:95%CI:0.561-0.774), 0.686(MLR:95%CI:0.571-0.802), and 0.771(combination:95%CI:0.674-0.869), respectively (**Figure 3C**). Indeed, the AUCs of inflammatory biomarkers were higher than clinical features [age (AUC:0.642, 95%CI:0.544-0.739), smoking (AUC:0.655, 95%CI:0.547-0.764), drinking (AUC:0.588, 95%CI:0.471-0.705), bronchiectasia (AUC:0.668, 95%CI:0.557-0.779), pulmonary cavity (AUC:0.663, 95%CI:0.567-0.760), pleural effusion (AUC:0.639, 95%CI:0.522-0.755)] (**Figure 3D**).

## 4 Discussion

Overall, we observed 24-month mortality rates of 12.0% in the training set and 20.5% in the validation set for patients with RR/MDR-TB. The rates in this study were higher than the that reported in previous literature for TB patients including both RR/MDR-TB and DS-TB (Khazaei et al., 2005; Martínez-Rodríguez et al., 2016), as explained by a higher mortality rate in RR/MDR-TB than DS-TB (Chung-Delgado et al., 2015).

Previous studies have elucidated in TB patients the prognostic value of various factors such as age, sex, residence, smoking, first sputum result and HIV status (Limenih and Workie, 2019; Xie et al., 2020; Carter et al., 2021). It is crucial for TB patients to find valid mortality predictors, especially in RR/MDR-TB patients, to help individualize management and reduce mortality. Recently, increasing attention have been paid to the interaction between TB and inflammation. The release of inflammatory factors and the formation of an inflammatory micro-environment provide a favorable environment for tissue destruction, the spread of bacteria (Amaral et al., 2021), and development and progression of TB (Krug et al., 2021). To date, the prognostic value of inflammatory biomarkers in TB patients has been rarely reported. A retrospective clinical study found that high-sensitivity C-reactive protein could predict early mortality in TB patients co-infected with HIV (Ciccacci et al., 2021). In our study, high levels of CAR, CPR, CLR, NLR, PLR, and MLR were risk factors for mortality in RR/MDR-TB patients.

C-reactive protein is most commonly used non-specific inflammatory biomarker in clinical practice, which can reflect the body’s inflammatory status. Albumin and prealbumin were visceral proteins valuable to assess the nutritional status of the body. Previous studies have shown that albumin (Sheinenzon et al., 2021) and prealbumin (Dennis et al., 2008) are negatively correlated with inflammatory factors such as CRP and have anti-inflammatory and antioxidant effects. Similarly, lymphocytes were also proven to have an effect on immunity and chronic inflammation (Kurtul et al., 2014). A previous study suggested that lymphocytopenia represents a state of critical inflammation (Li et al., 2021). Therefore, CAR, CPR, and CLR could better represent the body’s inflammatory and nutritional status. In this study, we explored their prognostic value in RR/MDR-TB patients. Our study found that patients with high levels of CAR, CPR, and CLR had a low survival rate. To date, this is the first study to report that RR/MDR-TB patients with high levels of CAR, CPR and CLR have a low survival rate. Currently, CAR (Karabağ et al., 2019; Karimi et al., 2021; Keinänen et al., 2021), CPR (Xie et al., 2011; Karimi et al., 2021; Maruyama et al., 2022), and CLR (Yang et al., 2020; Ko et al., 2021; Taniai et al., 2021) were reported to be prognostic factors for COVID-19, acute renal injury, and malignant tumor. In TB, previous studies have focused on the diagnostic value of inflammatory markers. For example, high-sensitivity C-reactive protein to lymphocyte ratio (HSCLR) was proven to be an important biomarker for distinguishing active PTB from inactive PTB (Yu et al., 2022b). Similarly, CLR can be used to predict the prevalence of T2DM in patients with active PTB (Yu et al., 2022a). To our best knowledge, the current study is the first to explore the correlation between CPR and prognosis of TB.

NLR, PLR, and MLR were newly discovered inflammatory markers in recent years. They are simple, easy to measure, and relatively stable compared with other inflammatory markers, such as interleukins. Previous studies have suggested that NLR, PLR, and MLR are diagnostic markers TB infection. NLR could identify TB in HIV-positive patients (Miyahara et al., 2019), and distinguish PTB from bacterial community-acquired pneumonia (Berhane et al., 2019). PLR was found to be a useful marker for identifying TB infection in COPD patients (Chen et al., 2016). MLR could be used to differentiate spinal TB from non-spinal TB (Chen et al., 2022). Interestingly, several studies have shown that high MLR could predict the severity of TB (Buttle et al., 2021; Chen et al., 2022). In this study, higher mortalities were observed in high NLR, PLR and MLR groups in RR/MDR-TB patients.

In addition, we explored the prognostic value of clinical characteristics in patients with RR/MDR-TB. We observed lower survival rates in the high age, smoking and bronchiectasia groups, which is similarly consistent with previous studies (Bates et al., 2007; Lin et al., 2007; Martínez-Rodríguez et al., 2016; Heunis et al., 2017; Wang et al., 2018). Frequent Comorbidities in older RR/MDR-TB patients, such as cardiovascular diseases, diabetes mellitus, and malignancies, could explain why the high age group had a higher mortality (Pardeshi, 2009). In addition, previous literature has already explained the mechanisms by which smoking affects the prognosis of TB patients. Nicotine can reduce the release of TNF-α, a key cytokine involved in granuloma formation by macrophages through α-7 nicotinic receptors (Silva et al., 2018). Interestingly, results from limited literature have examined the prognostic effect of bronchiectasia on RR/MDR-TB patients. Scholars found that bronchiectasia severity index could predict the mortality in post-tuberculosis bronchiectasia (Wang et al., 2018). TB infection is one of the leading causes of bronchiectasia in Asia-Pacific region. Pseudomonas aeruginosa, once co-infected with TB, may result in a reduced microbiota diversity, a disease progression and a poor prognosis (Dicker et al., 2021).

Based on these findings, the prognosis of RR/MDR-TB patients depend on multiple factors. The results of this study showed that inflammatory biomarkers, age, smoking and bronchiectasia, especially inflammatory biomarkers, were important prognostic factors in RR/MDR-TB, Inflammatory biomarkers had superior prognostic value compared to clinical characteristics in both the training and validation sets as evidenced by higher AUC. In addition, inflammatory biomarkers are easily and conveniently available indicators, and may provide physicians with more information and better guidance to improve the prognosis of RR/MDR-TB patients. CAR, CPR, CLR, NLR, PLR and MLR are good indicators of systemic inflammation (Llop-Talaveron et al., 2018; Okugawa et al., 2020; Jiang et al., 2021), and CAR and CPR can also reflect nutritional status of individuals (Llop-Talaveron et al., 2018). They can be easily and inexpensively calculated by the blood routine and biochemical test before ATT, independent of dehydration or fluid retention. The current study found that high levels of CAR, CPR, CLR, NLR, PLR, and MLR are essential prognostic factors for RR/MDR-TB patients, suggesting the importance for optimizing nutritional status, and improving hyperinflammatory status, thus minimizing mortality rate in RR/MDR-TB patients.

In conclusion, CAR, CPR, CLR, NLR, PLR, and MLR are economical and direct prognostic indicators valuable for assessing inflammation and nutrition status in patients with RR/MDR-TB. RR/MDR-TB patients with higher levels of CAR, CPR, CLR, NLR, PLR, and MLR indicate a higher mortality risk. Therefore, more attention should be paid to inflammatory biomarkers s in clinical practice.

## Data availability statement

The original contributions presented in the study are included in the article/**Supplementary Material**. Further inquiries can be directed to the corresponding authors.

## Ethics statement

The studies involving human participants were reviewed and approved by the Ethic Committee of Wuhan Jinyintan Hospital (KY-2022-06.01). Written informed consent to participate in this study was provided by the participants’ legal guardian/next of kin.

## Author contributions

JY, QN, and DS conceived the study. QY, SH, QN, and HL collected samples and clinical data. QY, QN, SH, and HL were responsible for statistical analysis. QY and QN interpreted the data and wrote the manuscript. All authors contributed to the article and approved the submitted version.
