# Supplementary material for: The survival analysis of rifampicin/multidrug-resistant tuberculosis patients based on the levels of inflammatory biomarkers: a retrospective cohort study
Source: Front Cell Infect Microbiol. 2023 May 1;13:1118424. doi: 10.3389/fcimb.2023.1118424 (PMC10183571; doi:10.3389/fcimb.2023.1118424)
Supplement: Supplementary file 1 [file DataSheet_1.docx]

**Definitions^1,2^**

**RR-TB:** TB with resistance to rifampin.

**MDR-TB:** TB with resistance to at least isoniazid and rifampin.

**Cure:** Treatment completed as recommended by the national policy without evidence of failure AND three or more consecutive cultures taken at least 30 days apart are negative after the intensive phase.

**Completed treatment:** Treatment completed as recommended by the national policy without evidence of failure BUT no record that three or more consecutive cultures taken at least 30 days apart are negative after the intensive phase.

**Treatment failure:** Treatment terminated or need for permanent regimen change of at least

two anti-TB drugs because of:

− lack of conversionb by the end of the intensive phasea , *or*

− bacteriological reversionb in the continuation phase after conversionb to negative, *or*

− evidence of additional acquired resistance to fluoroquinolones or

second-line injectable drugs, *or*

− adverse drug reactions.

Death: A patient who dies for any reason during the course of treatment.

**Loss to follow-up:** A patient whose treatment was interrupted for 2 consecutive months or

more.

**Reference**

1. World Health O. *WHO consolidated guidelines on drug-resistant tuberculosis treatment*. World Health Organization; 2019.

2. World Health O. *Definitions and reporting framework for tuberculosis – 2013 revision: updated December 2014 and January 2020*. World Health Organization; 2013.
