# Supplementary material for: The survival analysis of rifampicin/multidrug-resistant tuberculosis patients based on the levels of inflammatory biomarkers: a retrospective cohort study
Source: Front Cell Infect Microbiol. 2023 May 1;13:1118424. doi: 10.3389/fcimb.2023.1118424 (PMC10183571; doi:10.3389/fcimb.2023.1118424)
Supplement: Supplementary file 2 [file Image_1.pdf]

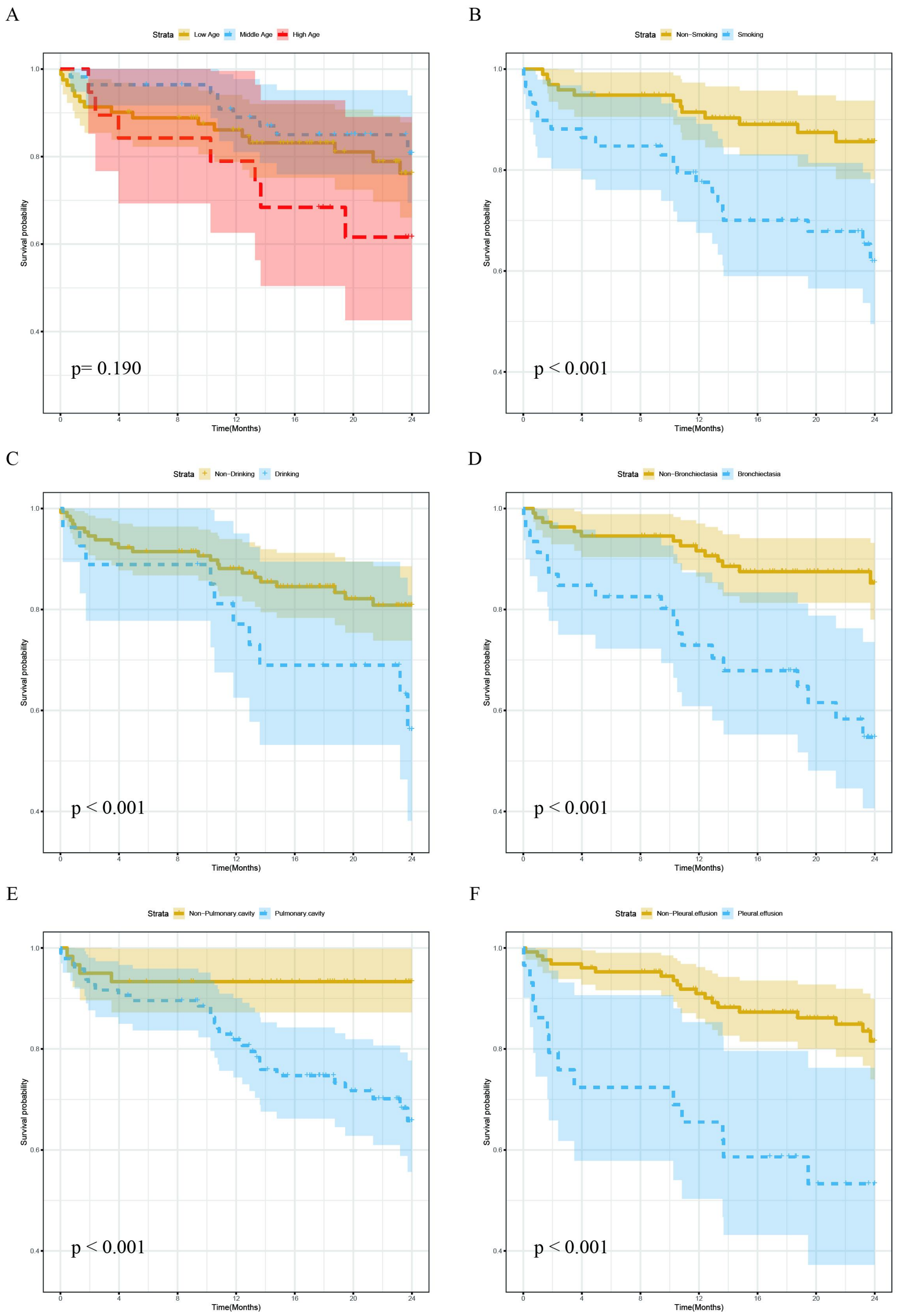


**Supplementary Figure 1.** 24-month survival rate of RR/MDR-TB patients according to the levels of clinical characteristics in validation set.

**Note:** (A)The survival analysis according to age. (B)The survival analysis according to smoking. (C)The survival analysis according to Drinking. (D)The survival analysis according to Bronchiectasia. (E)The survival analysis according to Pulmonary cavity. (F)The survival analysis according to Pleural effusion.
