# Supplementary material for: The survival analysis of rifampicin/multidrug-resistant tuberculosis patients based on the levels of inflammatory biomarkers: a retrospective cohort study
Source: Front Cell Infect Microbiol. 2023 May 1;13:1118424. doi: 10.3389/fcimb.2023.1118424 (PMC10183571; doi:10.3389/fcimb.2023.1118424)
Supplement: Supplementary file 3 [file Image_2.pdf]

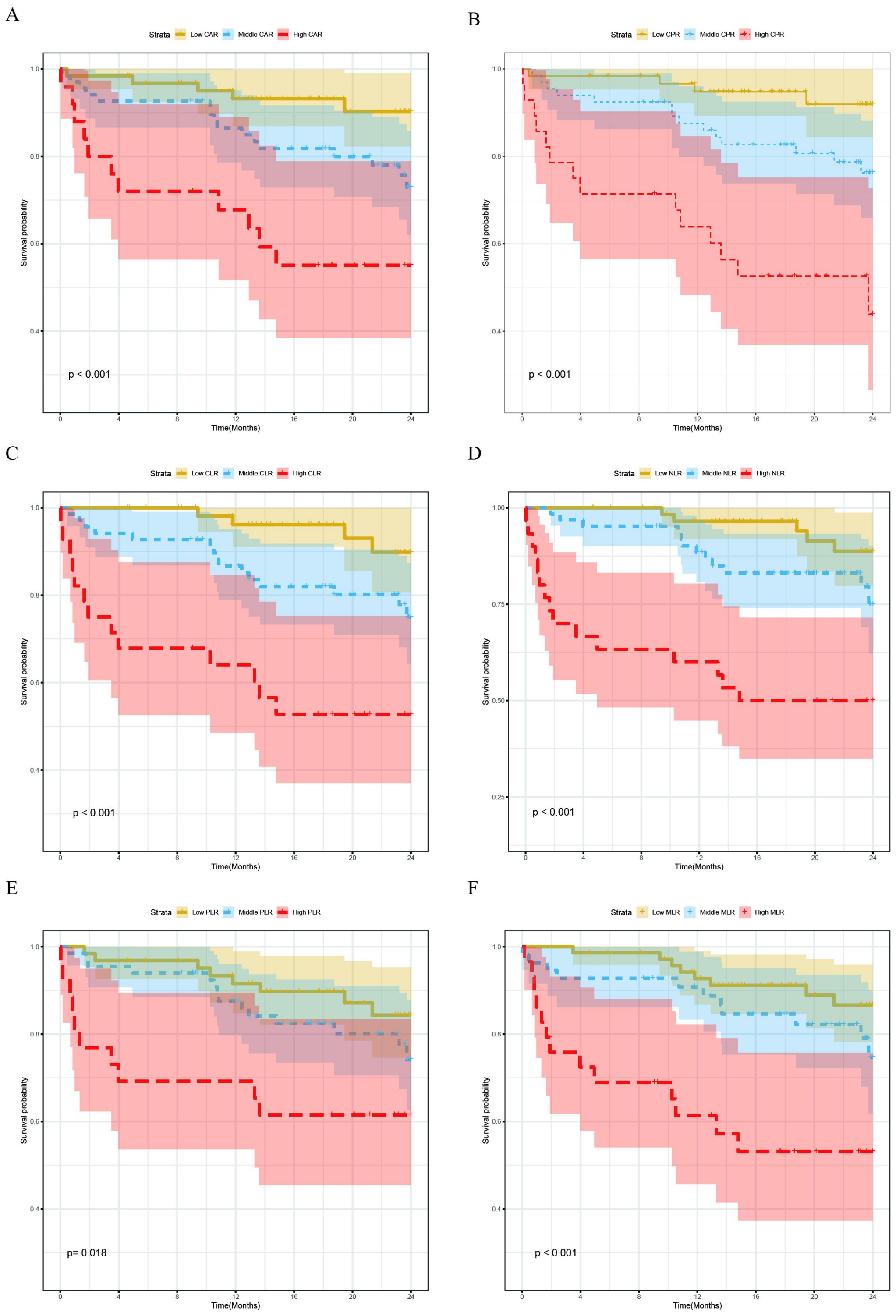

Figure 2. 24-month survival rate of RR/MDR-TB patients according to risk stratification of inflammatory biomarkers in validation set.

Note: (A)The survival analysis according to risk stratification of CAR. (B)The survival analysis according to risk stratification of CPR. (C)The survival analysis according to risk stratification of CLR. (D)The survival analysis according to risk stratification of NLR. (E)The survival analysis according to risk stratification of PLR. (F)The survival analysis according to risk

stratification of MLR.
