# Supplementary material for: The survival analysis of rifampicin/multidrug-resistant tuberculosis patients based on the levels of inflammatory biomarkers: a retrospective cohort study
Source: Front Cell Infect Microbiol. 2023 May 1;13:1118424. doi: 10.3389/fcimb.2023.1118424 (PMC10183571; doi:10.3389/fcimb.2023.1118424)
Supplement: Supplementary file 4 [file Table_1.docx]

**Supplementary Table 1 Comparison of clinical characteristics among high/middle/low CAR group in RR/MDR-TB patients**

| **Characteristic** | **Low CAR group(N=174)** | **Median CAR group(N=122)** | **High CAR group(N=52)** | **P value** |
| --- | --- | --- | --- | --- |
| Age （Mean±SD） | 41.02±14.64 | 44.28±16.29 | 45.23±15.65 | 0.095 |
| **Gender** |  |  |  |  |
| Female | 56 | 21 | 7 |  |
| Male | 118 | 101 | 45 | 0.002 |
| **Treatment outcome** |  |  |  |  |
| Cure | 71 | 40 | 11 |  |
| Treatment completed | 69 | 40 | 10 |  |
| Lost to follow-up | 5 | 4 | 6 |  |
| Failure | 21 | 21 | 8 |  |
| Death | 8 | 17 | 17 | 0 |
| **Drug sensitivity** |  |  |  |  |
| RR-TB | 34 | 25 | 10 |  |
| MDR-TB | 61 | 29 | 18 |  |
| XDR-TB | 79 | 68 | 24 | 0.297 |
| **Smoking** | 64 | 56 | 24 | 0.219 |
| **Drinking** | 21 | 22 | 9 | 0.320 |
| **Pulmonary imaging** |  |  |  |  |
| **Bronchiectasia** | 52 | 45 | 30 | 0.001 |
| **Pulmonary cavity** | 108 | 86 | 39 | 0.129 |
| **Pleural effusion** | 10 | 28 | 16 | <0.001 |
| **Underlying condition or illness** |  |  |  |  |
| **Diabetes mellitus** | 22 | 23 | 7 | 0.320 |
| **HIV infection** | 3 | 4 | 4 | 0.097 |

**Supplementary Table 2 Comparison of clinical characteristics among high/middle/low CPR group in RR/MDR-TB patients**

| Characteristic | Low CAR group(N=174) | Median CAR group(N=122) | High CAR group(N=52) | P value |
| --- | --- | --- | --- | --- |
| Age （Mean±SD） | 41.44±14.76 | 43.94±16.24 | 44.63±15.67 | 0.253 |
| **Gender** |  |  |  |  |
| Female | 52 | 25 | 7 |  |
| Male | 122 | 97 | 45 | 0.027 |
| **Treatment outcome** |  |  |  |  |
| Cure | 70 | 42 | 10 |  |
| Treatment completed | 69 | 38 | 12 |  |
| Lost to follow-up | 5 | 5 | 5 |  |
| Failure | 22 | 19 | 9 |  |
| Death | 8 | 18 | 16 | <0,001 |
| **Drug sensitivity** |  |  |  |  |
| RR-TB | 33 | 25 | 11 |  |
| MDR-TB | 61 | 28 | 19 |  |
| XDR-TB | 80 | 69 | 22 | 0.175 |
| **Smoking** | 67 | 54 | 23 | 0.553 |
| **Drinking** | 22 | 22 | 8 | 0.439 |
| **Pulmonary imaging** |  |  |  |  |
| **Bronchiectasia** | 54 | 46 | 27 | 0.022 |
| **Pulmonary cavity** | 108 | 88 | 37 | 0.152 |
| **Pleural effusion** | 11 | 28 | 15 | <0,001 |
| **Underlying condition or illness** |  |  |  |  |
| **Diabetes mellitus** | 22 | 23 | 7 | 0.320 |
| **HIV infection** | 3 | 5 | 3 | 0.262 |

**Supplementary Table 3 Comparison of clinical characteristics among high/middle/low CLR group in RR/MDR-TB patients**

| Characteristic | Low CAR group(N=174) | Median CAR group(N=122) | High CAR group(N=52) | P value |
| --- | --- | --- | --- | --- |
| Age （Mean±SD） | 40.74±14.72 | 44.55±16.10 | 45.56±15.64 | 0.042 |
| **Gender** |  |  |  |  |
| Female | 56 | 20 | 8 |  |
| Male | 118 | 102 | 44 | 0.002 |
| **Treatment outcome** |  |  |  |  |
| Cure | 72 | 40 | 10 |  |
| Treatment completed | 68 | 39 | 12 |  |
| Lost to follow-up | 4 | 6 | 5 |  |
| Failure | 23 | 18 | 9 |  |
| Death | 7 | 19 | 16 | <0,001 |
| **Drug sensitivity** |  |  |  |  |
| RR-TB | 30 | 27 | 12 |  |
| MDR-TB | 62 | 29 | 17 |  |
| XDR-TB | 82 | 66 | 23 | 0.239 |
| **Smoking** | 63 | 58 | 23 | 0.135 |
| **Drinking** | 22 | 21 | 9 | 0.485 |
| **Pulmonary imaging** |  |  |  |  |
| **Bronchiectasia** | 52 | 49 | 26 | 0.018 |
| **Pulmonary cavity** | 108 | 89 | 36 | 0.137 |
| **Pleural effusion** | 9 | 29 | 16 | 0 |
| **Underlying condition or illness** |  |  |  |  |
| **Diabetes mellitus** | 21 | 24 | 7 | 0.186 |
| **HIV infection** | 3 | 4 | 4 | 0.097 |
|  |  |  |  |  |

**Supplementary Table 4 Comparison of clinical characteristics among high/middle/low NLR group in RR/MDR-TB patients**

| Characteristic | Low CAR group(N=174) | Median CAR group(N=122) | High CAR group(N=52) | P value |
| --- | --- | --- | --- | --- |
| Age （Mean±SD） | 41.07±14.00 | 43.73±15.91 | 46.35±15.31 | 0.754 |
| **Gender** |  |  |  |  |
| Female | 53 | 23 | 8 |  |
| Male | 121 | 99 | 44 | 0.020 |
| **Treatment outcome** |  |  |  |  |
| Cure | 70 | 42 | 10 |  |
| Treatment completed | 69 | 40 | 10 |  |
| Lost to follow-up | 6 | 2 | 7 |  |
| Failure | 23 | 17 | 10 |  |
| Death | 6 | 21 | 15 | <0,001 |
| **Drug sensitivity** |  |  |  |  |
| RR-TB | 31 | 25 | 13 |  |
| MDR-TB | 59 | 36 | 13 |  |
| XDR-TB | 84 | 61 | 26 | 0.681 |
| **Smoking** | 64 | 53 | 27 | 0.128 |
| **Drinking** | 19 | 25 | 8 | 0.075 |
| **Pulmonary imaging** |  |  |  |  |
| **Bronchiectasia** | 46 | 52 | 29 | <0,001 |
| **Pulmonary cavity** | 109 | 88 | 36 | 0.216 |
| **Pleural effusion** | 17 | 25 | 12 | 0.011 |
| **Underlying condition or illness** |  |  |  |  |
| **Diabetes mellitus** | 26 | 23 | 3 | 0.086 |
| **HIV infection** | 4 | 3 | 4 | 0.128 |

**Supplementary Table 5 Comparison of clinical characteristics among high/middle/low PLR group in RR/MDR-TB patients**

| Characteristic | Low CAR group(N=174) | Median CAR group(N=122) | High CAR group(N=52) | P value |
| --- | --- | --- | --- | --- |
| Age （Mean±SD） | 42.02±15.17 | 42.39±15.13 | 46.31±16.87 | 0.686 |
| **Gender** |  |  |  |  |
| Female | 46 | 28 | 10 |  |
| Male | 128 | 94 | 42 | 0.527 |
| **Treatment outcome** |  |  |  |  |
| Cure | 68 | 42 | 12 |  |
| Treatment completed | 63 | 42 | 14 |  |
| Lost to follow-up | 3 | 7 | 5 |  |
| Failure | 31 | 12 | 7 |  |
| Death | 9 | 19 | 14 | <0,001 |
| **Drug sensitivity** |  |  |  |  |
| RR-TB | 35 | 25 | 9 |  |
| MDR-TB | 56 | 36 | 16 |  |
| XDR-TB | 83 | 61 | 27 | 0.969 |
| **Smoking** | 73 | 46 | 25 | 0.435 |
| **Drinking** | 24 | 21 | 7 | 0.682 |
| **Pulmonary imaging** |  |  |  |  |
| **Bronchiectasia** | 50 | 54 | 23 | 0.011 |
| **Pulmonary cavity** | 116 | 82 | 35 | 0.993 |
| **Pleural effusion** | 11 | 27 | 16 | <0,001 |
| **Underlying condition or illness** |  |  |  |  |
| **Diabetes mellitus** | 32 | 16 | 4 | 0.129 |
| **HIV infection** | 3 | 44 | 4 | 0.097 |

**Supplementary Table 6 Comparison of clinical characteristics among high/middle/low MLR group in RR/MDR-TB patients**

| Characteristic | Low CAR group(N=174) | Median CAR group(N=122) | High CAR group(N=52) | P value |
| --- | --- | --- | --- | --- |
| Age （Mean±SD） | 41.95±15.04 | 42.75±15.36 | 45.71±16.89 | 0.620 |
| **Gender** |  |  |  |  |
| Female | 57 | 20 | 7 |  |
| Male | 117 | 102 | 45 | 0.001 |
| **Treatment outcome** |  |  |  |  |
| Cure | 66 | 42 | 14 |  |
| Treatment completed | 67 | 35 | 17 |  |
| Lost to follow-up | 10 | 5 | 0 |  |
| Failure | 25 | 17 | 8 |  |
| Death | 6 | 23 | 13 | <0,001 |
| **Drug sensitivity** |  |  |  |  |
| RR-TB | 32 | 25 | 12 |  |
| MDR-TB | 61 | 34 | 13 |  |
| XDR-TB | 81 | 63 | 27 | 0.58 |
| **Smoking** | 62 | 56 | 26 | 0.082 |
| **Drinking** | 18 | 24 | 10 | 0.055 |
| **Pulmonary imaging** |  |  |  |  |
| **Bronchiectasia** | 52 | 49 | 26 | 0.018 |
| **Pulmonary cavity** | 109 | 92 | 32 | 0.048 |
| **Pleural effusion** | 15 | 19 | 20 | <0,001 |
| **Underlying condition or illness** |  |  |  |  |
| **Diabetes mellitus** | 25 | 22 | 5 | 0.346 |
| **HIV infection** | 4 | 4 | 3 | 0.453 |

**Supplementary Table 7 Cox proportion risk regression analysis in RR/MDR-TB patients in the training set.**

| Parameters | P value | OR | 95%CI | |
| --- | --- | --- | --- | --- |
|  |  |  | down | Up |
| CAR | <0,001 | 1.343 | 1.176 | 1.534 |
| CPR | <0,001 | 1.962 | 1.478 | 2.604 |
| CLR | <0,001 | 1.007 | 1.004 | 1.009 |
| NLR | <0,001 | 1.156 | 1.107 | 1.206 |
| PLR | <0,001 | 1.002 | 1.001 | 1.003 |
| MLR | <0,001 | 5.825 | 2.801 | 12.116 |
| Bronchiectasia | <0,001 | 3.726 | 1.85 | 7.503 |
| Pulmonary cavity | 0.004 | 4.727 | 1.657 | 13.482 |
| Pleural effusion | <0,001 | 3.739 | 1.843 | 7.585 |
| Smoking | 0.003 | 2.961 | 1.447 | 6.059 |
| Drinking | 0.035 | 2.239 | 1.06 | 4.729 |
| Age | 0.015 | 1.028 | 1.005 | 1.052 |

**Supplementary Table 8 The cut-off value of inflammatory biomarkers.**

| Inflammatory biomarkers | Cut-off value | |
| --- | --- | --- |
|  | Median | 85th percentile |
| CAR | 0.6003 | 2.514 |
| CPR | 0.1555 | 0.8678 |
| CLR | 18.6559 | 87.3806 |
| NLR | 3.5557 | 7.9795 |
| PLR | 213.7319 | 395.4583 |
| MLR | 0.4101 | 0.7732 |
| Age | 44 | 60 |
